# Accurate OPM–MEG Co-Registration via Magnetic Dipole-Based Sensor Localization with Rigid Coil Structures and Optical Direction Constraints

**DOI:** 10.3390/bioengineering12121370

**Published:** 2025-12-16

**Authors:** Weinan Xu, Wenli Wang, Fuzhi Cao, Nan An, Wen Li, Baosheng Wang, Chunhui Wang, Xiaolin Ning, Ying Liu

**Affiliations:** 1 School of Instrumentation and Optoelectronic Engineering, Beihang University, Beijing 100191, China; xwnan@buaa.edu.cn (W.X.); zb1917006@buaa.edu.cn (W.W.); caofuzhi@buaa.edu.cn (F.C.); liwen1997@buaa.edu.cn (W.L.); chunhuiwang@buaa.edu.cn (C.W.); 2 National Institute of Extremely Weak Magnetic Field Infrastructure, Hangzhou 310028, China; annan@buaa.edu.cn (N.A.); wangbsh1@shanghaitech.edu.cn (B.W.); 3 Hangzhou Lingci Medical Equipment Co., Ltd., Hangzhou 310057, China; 4 Hefei National Laboratory, Hefei 230088, China

**Keywords:** OPM–MEG, sensor co-registration, magnetic dipole, rigid coil structure (RCS), optical orientation constraint, single-axis OPM

## Abstract

Accurate co-registration between on-scalp Optically Pumped Magnetometer (OPM)–Magnetoencephalography (MEG) sensors and anatomical Magnetic Resonance Imaging (MRI) remains a critical bottleneck restricting the spatial fidelity of source localization. Optical Scanning Image (OSI) methods can provide high spatial accuracy but depend on surface visibility and cannot directly determine the internal sensitive point of each OPM sensor. Coil-based magnetic dipole localization, in contrast, targets the sensor’s internal sensitive volume and is robust to occlusion, yet its accuracy is affected by coil fabrication imperfections and the validity of the dipole approximation. To integrate the complementary advantages of both approaches, we propose a hybrid co-registration framework that combines Rigid Coil Structures (RCS), magnetic dipole-based sensor localization, and optical orientation constraints. A complete multi-stage co-registration pipeline is established through a unified mathematical formulation, including MRI–OSI alignment, OSI–RCS transformation, and final RCS–sensor localization. Systematic simulations are conducted to evaluate the accuracy of the magnetic dipole approximation for both cylindrical helical coils and planar single-turn coils. The results quantify how wire diameter, coil radius, and turn number influence dipole model fidelity and offer practical guidelines for coil design. Experiments using 18 coils and 11 single-axis OPMs demonstrate positional accuracy of a few millimeters, and optical orientation priors suppress dipole-only orientation ambiguity in unstable channels. To improve the stability of sensor orientation estimation, optical scanning of surface markers is incorporated as a soft constraint, yielding substantial improvements for channels that exhibit unstable results under dipole-only optimization. Overall, the proposed hybrid framework demonstrates the feasibility of combining magnetic and optical information for robust OPM–MEG co-registration.

## 1. Introduction

Magnetoencephalography (MEG) is a non-invasive neuroimaging technique capable of recording neuronal activity in the human brain with high temporal and spatial resolution. It has been widely applied in neuroscience research, clinical diagnosis of neurological disorders, and brain–computer interface development [1,2,3]. Traditional low-temperature superconducting quantum interference device (Low-Tc SQUID) systems have achieved mature and stable performance after decades of development [4]. However, their dependence on cryogenic cooling and rigid helmet structures results in high maintenance costs, limited subject comfort, and a requirement of fixed head position during acquisition, which restricts studies of naturalistic behavior and pediatric populations [5,6].

In recent years, optically pumped magnetometers (OPMs) have enabled the rapid development of a new generation of wearable MEG systems. OPM sensors operate at room temperature and can be placed directly on the scalp, providing substantially increased signal magnitude and greater spatial flexibility [7,8,9]. Nevertheless, OPM-based systems typically employ flexible cap structures, and inter-subject variability in head shape causes uncontrolled sensor positions and orientations. As a result, achieving high-accuracy sensor registration has become a critical bottleneck for OPM–MEG systems. Registration errors directly affect the spatial precision of source localization and compromise the reliability of subsequent imaging results [10,11]. Recent developments in OPM technology have enabled various biomedical and magnetic-field–sensing applications, such as magnetic particle detection and flow measurement, which further demonstrate the feasibility of high-sensitivity magnetic field detection using OPM systems [12,13].

Existing registration methods can be broadly categorized into two classes. The first class consists of electromagnetic coil–based magnetic dipole modeling, in which powered coils are attached to specific scalp locations and the resulting magnetic fields are measured by the sensors to determine their spatial positions [14,15].

The second class relies on optical scanning and morphological matching, where the head surface is acquired by structured-light or laser scanning and subsequently registered to the MRI-derived facial model using the iterative closest point (ICP) algorithm [16,17]. Coil-based methods can determine the internal sensitive point of each sensor but are affected by placement errors, cable tension, and deformation of flexible caps. Optical scanning methods provide extremely high geometric accuracy but cannot determine the sensors’ true sensitive points. Each method therefore offers clear advantages but also exhibits characteristic limitations.

For accurate mapping of neuronal activity, the spatial relationship between neuronal sources and MEG sensors must be precisely established. This requires an accurate transformation between two coordinate systems: the MEG coordinate system, defined by the OPM array, and the brain coordinate system, obtained from magnetic resonance imaging (MRI). The MRI coordinate frame is derived from anatomical scans and cortical surface extraction using tools such as FreeSurfer [18]. Consequently, precise co-registration between these coordinate systems is essential, as registration errors directly propagate to inaccuracies in source localization [19,20].

In conventional SQUID–MEG systems, head–sensor registration is typically achieved using head position indicator (HPI) coils together with electromagnetic or optical tracking systems [21,22,23,24,25]. These approaches perform well under rigid helmet conditions and can achieve positional accuracy better than 5 mm, or even 3 mm when supported by high-precision structured-light scanning. For flexible MEG caps, however, registration remains a major challenge. Existing approaches often combine HPI coils with optical trackers [26,27], but coil placement and tracking errors introduce significant random deviations [28]. Other methods attempt to segment and identify sensor regions directly from optical point clouds [29], yet cable occlusion and surface reflections reduce their robustness.

Recent studies have explored hybrid strategies combining optical scanning and electromagnetic coils, such as the Sensor Position Indicator (SPI) technique [30], which captures facial geometry and coil locations via structured-light scanning. Although this approach benefits from the geometric accuracy of optical scanning, practical implementations suffer from coil deformation and cable-induced displacement between scanning and measurement sessions. Detachable-cable OPM sensors [31] allow orientation recording during scanning but reattachment often introduces unpredictable shifts. At present, no flexible-cap–based coil registration method has been successfully applied to OPM–MEG systems, and most related work remains limited to SQUID-based validation [32,33].

In this study, we address the current bottleneck of accurate sensor registration in OPM–MEG systems by combining the complementary strengths of magnetic coil–based localization and optical geometric constraints. Optical scanning provides high spatial accuracy but depends on surface visibility and cannot determine each sensor’s internal sensitive point. Magnetic dipole–based localization, in contrast, directly estimates the internal sensitive volume and remains robust under occlusion or head motion, although its accuracy is inherently influenced by coil fabrication imperfections, hardware noise, and the validity of the dipole approximation. To integrate these complementary advantages, we propose a hybrid co-registration framework based on rigid coil structures (RCS) [34], magnetic dipole fitting, and optical direction constraints.

The main contributions of this work are fourfold.

1. We develop a complete MEG–MRI co-registration pipeline that incorporates MRI–OSI, OSI–RCS, and RCS–sensor transformations within a unified mathematical framework suitable for wearable OPM arrays;
2. We systematically evaluate the magnetic dipole approximation for both cylindrical helical coils and planar single-turn coils, revealing how coil geometry—including wire diameter, turn number, and radius—affects dipole fitting accuracy and providing quantitative guidelines for coil design;
3. We perform real-world experiments using 18 coils and 11 OPM sensors to validate the end-to-end registration performance of the RCS-based system.
4. We introduce an optical orientation constraint to stabilize sensor direction estimation, demonstrating substantial improvements in orientation accuracy while preserving the robustness of coil-based localization.

Overall, this study provides both theoretical and experimental evidence supporting a hybrid OPM–MEG co-registration strategy. Although the absolute accuracy of our current hardware implementation is constrained by coil fabrication tolerances and device limits, the proposed framework offers practical design references and highlights promising pathways for future improvements, including higher-precision coils, PCB-based structures, and enhanced sensor calibration methods.

## 2. Materials and Methods

### 2.1. System Overview

A sensor co-registration system for optically pumped magnetometer–magnetoencephalography (OPM–MEG) based on a magnetic dipole–equivalent coil model was developed in this study. The proposed system integrates electromagnetic and optical modalities to achieve high-precision spatial localization of OPM sensors relative to the subject’s head.

The overall framework consists of three primary components:

1. A rigid coil structure (RCS) carrying an array of electromagnetic coils;
2. A flexible MEG cap accommodating multiple OPM sensors;
3. An optical scanning subsystem for acquiring 3D geometric information.

The registration procedure begins with a structured-light scan to obtain the three-dimensional geometry of both the coil array and the subject’s head. The acquired point-cloud data are then processed to establish the geometric correspondence between the RCS and head surface. Subsequently, a magnetic dipole–equivalent modeling and optimization algorithm is employed to estimate each OPM sensor’s spatial position and orientation in the MRI coordinate space.

This hybrid registration system aims to provide a unified spatial mapping between the OPM array and the MRI-derived anatomical model. By combining the geometric precision of optical scanning with the physical accuracy of electromagnetic modeling, the framework establishes a robust foundation for accurate source localization in OPM–MEG studies.

#### 2.1.1. Rigid Coil Structure (RCS)

The flexible MEG cap and OPM sensor array were designed to integrate seamlessly with the rigid coil structure (RCS) developed in our previous work [34]. While the structural details and material composition of the RCS were described in [34], the present study focuses on the sensor configuration and its spatial relationship with the coil array.

The flexible MEG cap follows a standard EEG layout and accommodates multiple optically pumped magnetometers (QuSpin Gen-II, QuSpin Inc., Louisville, CO, USA). Each OPM sensor is mounted in an individually designed slot that maintains stable mechanical contact with the scalp surface and preserves the sensor’s fixed orientation throughout the measurement process.

**Figure 1:**
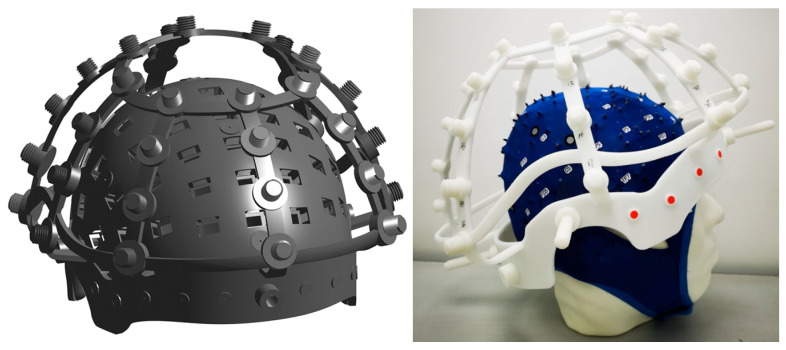
Design schematic and physical prototype of the RCS.

A total of 85 OPM sensor slots were distributed across the scalp region to cover major cortical areas, while 35 excitation coils were mounted on the RCS to serve as localization references. Figure 1 illustrates the RCS design model and its fabricated prototype. To facilitate optical alignment, 10 color-coded markers were positioned on the frontal section of the RCS, enabling precise spatial correspondence between the optical scanning coordinate system and the RCS reference frame.

Note that the RCS contains 35 coil mounting slots to accommodate different sensor layouts and potential full-head configurations. However, the localization algorithm does not require all coils to be activated simultaneously; it only uses the coils that are driven in a particular experiment.

#### 2.1.2. Coil Current Driver System

The coil current driver system consisted of a Keysight 33500B signal generator (Keysight Technologies, Santa Rosa, CA, USA) and a Stanford Research Systems CS580 current source (Sunnyvale, CA, USA). The signal generator produced a sinusoidal voltage waveform that served as a stable input for the current source, while the CS580 provided a low-noise alternating current to sequentially drive each excitation coil mounted on the RCS.

The driving frequency was selected based on the measured environmental magnetic noise to avoid overlap with the dominant power-line interference. This configuration ensured stable magnetic field generation and minimized external electromagnetic disturbances during the localization experiments.

In our experiments, the driving frequency for all excitation coils was chosen within the 15–30 Hz low-noise band identified through preliminary environmental spectral analysis. A driving frequency of 20 Hz was used in all measurements. Each coil was energized for 2.0 s, during which the OPM responses were continuously recorded. With 18 coils activated sequentially, the total magnetic-field acquisition time was approximately 36 s. Including the structured-light optical scan and subsequent data processing, the full hybrid co-registration procedure required approximately 2–3 min to complete.

#### 2.1.3. Structured Light Scanner

The three-dimensional structural data was acquired using the EinScan H structured light scanner (SHINING 3D Inc., Hangzhou, China). The scanner achieves a maximum measurement accuracy of 0.05 mm in white light mode with a working distance of 470 mm. Its operating principle involves projecting structured light patterns onto the object surface and reconstructing 3D point clouds based on pattern deformation. This scanning system was employed to capture spatial geometric information of the head and coil array, establishing a precise geometric foundation for subsequent registration processes.

### 2.2. The Registration Framework

**Figure 2:**
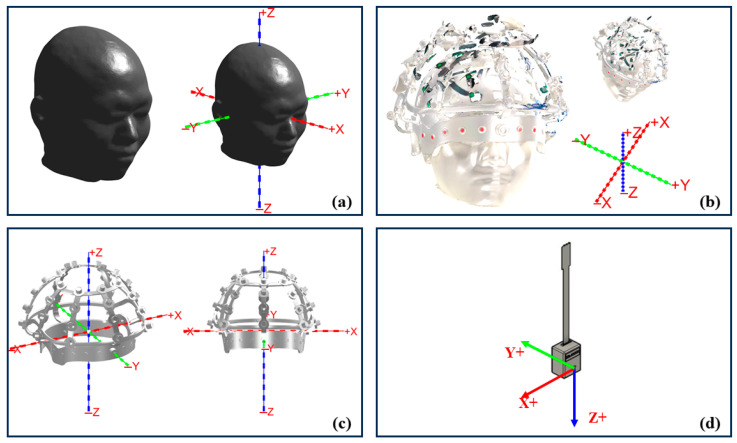
Four coordinate systems. (**a**) MRI head surface models; (**b**) Optical scanning (OSI) with sensor and coil markers; (**c**) Rigid Coil Structure (RCS) geometry; (**d**) Coordinate definition of a single OPM sensor.

To establish a unified spatial reference for OPM sensor localization, four coordinate systems were defined in this study, as illustrated in Figure 2.

Figure 2 shows the spatial arrangement of all 35 coils and highlights the subset of coils activated in the experimental configuration. This comparison illustrates the difference between the full simulation model and the reduced coil set used for experimental validation. Axes are shown in millimeters (mm).

#### 2.2.1. MRI Coordinate (MRI) System

The MRI coordinate system serves as the anatomical reference frame, within which the head surface and brain volume are represented. The coordinate system is defined with the *x*-axis pointing from posterior to anterior, the *y*-axis pointing from right to left, and the *z*-axis pointing from inferior to superior, consistent with the frame illustrated in Figure 2a.

#### 2.2.2. Optical Scanning Image (OSI) Coordinate System

The optical scanning coordinate system is automatically generated by the structured-light scanner during 3D surface acquisition. It defines the spatial positions of the scanned point clouds corresponding to both the RCS and the subject’s head.

#### 2.2.3. Rigid Coil Structure Coordinate (RCS) System

The RCS coordinate system is defined according to the 3D CAD model of the rigid coil array. Its origin is set at the geometric center of the structure, the *z*-axis is perpendicular to the frontal plane of the RCS, and the x–y plane coincides with the coil array surface. The coordinates of all excitation coils are expressed in this system.

#### 2.2.4. Single Sensor Coordinate System:

Each OPM sensor possesses an independent local coordinate system, within which the *z*-axis corresponds to the sensor’s magnetic sensitivity direction, and the x and y axes lie in the plane orthogonal to it. Determining each individual Sensor–MRI transformation constitutes the ultimate goal of the registration procedure.

### 2.3. Coil-Based MEG–MRI Co-Registration Process

#### 2.3.1. MRI-OSI

The MRI–OSI registration aligns the MRI-derived head surface with the optical 3D scan in order to place the optical point cloud into the anatomical MRI space. The head surface is first extracted from the MRI volume (using FreeSurfer or equivalent tools) to produce an MRI-based surface point set, while the structured-light scanner produces the OSI point-cloud. Because point-cloud fine-registration algorithms such as ICP are sensitive to initial alignment, a coarse pre-alignment is required to bring the two-point sets into approximate proximity before ICP refinement.

The point-cloud set extracted from the MRI head-surface image is denoted as $V_{MRI}$, and the point-cloud set obtained from the OSI image file is denoted as $V_{OSI}$. Among these, only the facial regions of the two point-clouds contain valid geometric features. Because of the coordinate information of the MRI model corresponds more clearly to human anatomical features, it is more convenient to extract the effective facial point-cloud features from the MRI dataset.

Given that the tip of the nose corresponds to the maximum value along the *y*-axis in the MRI coordinate frame, this property can be utilized to segment the nasal region. Accordingly, the subset of points around the nasal region is defined as $V_{MRI}^{sign}$, and the remaining subset of points is defined as $V_{MRI}^{other}$. Thus, we have Equation (1):

(1) $$V_{MRI}=V_{MRI}^{sign}+V_{MRI}^{other}$$

On the other hand, due to the uncertainty of the OSI coordinate system, a new auxiliary coordinate frame was reconstructed based on three anatomical landmarks—the nasion and the left and right preauricular points—to satisfy the requirements of coarse registration.

**Figure 3:**
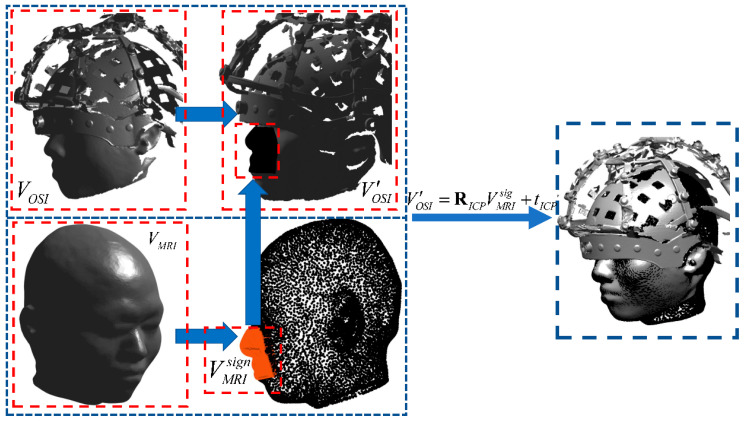
Workflow of the MRI–OSI co-registration.

By computing a rotation matrix $R_{OSI}$ and a translation vector $t_{OSI}$, the two point-cloud datasets can be approximately aligned in the same spatial region, as illustrated in Figure 3. This transformation serves as the initial positioning for subsequent fine registration. Thus, we have Equation (2):

(2) $$V_{OSI}^{\prime }=R_{OSI}V_{OSI}+t_{OSI}$$

After the coarse registration, the merged point-cloud set is denoted as $V_{OSI}^{\prime }$. The fine registration is performed between point-cloud sets $V_{MRI}^{sign}$ and $V_{OSI}^{\prime }$ using the Iterative Closest Point (ICP) algorithm. Through iterative computation based on the least-squares method, the optimal rotation matrix $R_{ICP}$ and translation vector $t_{ICP}$ between the two coordinate systems are determined such that Equation (3) is satisfied. The main computational process is shown in Equation (4).

(3) $$V_{OSI}^{\prime }=R_{ICP}V_{MRI}^{sign}+t_{ICP}$$

To refine the coarse alignment obtained from the magnetic-dipole fitting, an Iterative Closest Point (ICP) procedure is applied between the coil-model points and the reconstructed OSM points. Specifically, the coil-model point set is defined as $V_{MRI}^{sign}=\left[q_{MRI1}^{sign},q_{MRI2}^{sign},\ldots q_{MRIn}^{sign}\right]$. The corresponding set of OSM-reconstructed points is $V_{OSI}^{\prime }=\left[p_{OSI1}^{\prime },p_{OSI2}^{\prime },\ldots ,p_{OSIm}^{\prime }\right]$, where typically m≫n. These two-point sets serve as the inputs to the ICP refinement.

(4) $$\underset{R_{ICP},t_{ICP}}{\arg\min}{\sum_{i_{icp}=1}^{m}\left\|q_{MRIi_{icp}}^{sign}-R_{ICP}p_{OSIj_{icp}}^{\prime }-t_{ICP}\right\|}_{F}$$

Therefore, the transformation relationship between MRI and OSI is expressed by Equations (5) and (6).

(5) $$V_{OSI}=R_{OSI}^{-1}\left(R_{ICP}V_{MRI}+t_{ICP}-t_{OSI}\right)$$

(6) $$V_{OSI}=(R_{OSI}^{-1}R_{ICP})V_{MRI}+R_{OSI}^{-1}(t_{ICP}-t_{OSI})$$

Through this process, the final rotation matrix $R_{OSI}^{MRI}=R_{OSI}^{-1}R_{ICP}$ and translation vector $t_{OSI}^{MRI}=R_{OSI}^{-1}(t_{ICP}-t_{OSI})$ are obtained, defining the rigid-body transformation between the two coordinate systems.

#### 2.3.2. OSI-RCS

**Figure 4:**
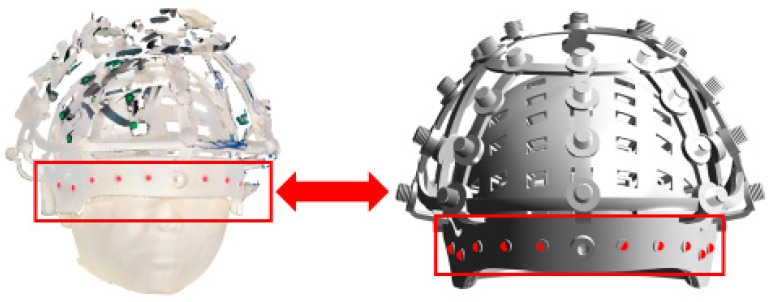
Ten color-coded fiducial markers of RCS.

The registration between OSI and RCS aims to determine the spatial transformation between the OSI coordinate system and the rigid coil structure coordinate system. Ten color-coded fiducial markers are uniformly distributed on the front surface of the RCS in Figure 4. These markers can be simultaneously recognized in both the optical scan and the RCS CAD model, thus serving as reliable reference points for coordinate alignment.

By extracting the three-dimensional coordinates of these ten markers, two corresponding point sets are obtained—denoted as $P_{OSI}=\left[p_{OSI1},p_{OSI2},\ldots p_{OSI10}\right]$ and $P_{RCS}=\left[p_{RCS1},p_{RCS2},\ldots ,p_{RCS10}\right]$. The objective of the OSI–RCS registration is to find the optimal rotation matrix $R_{OSI}^{RCS}$ and translation vector $t_{OSI}^{RCS}$ that minimize the mean squared error between the two point-sets, as expressed by Equation (7):

(7) $$\underset{R_{OSI}^{RCS},t_{OSI}^{RCS}}{\min}{\sum_{i=1}^{10}\left\|P_{RCSi}-\left(R_{OSI}^{RCS}P_{RCSi}+t_{OSI}^{RCS}\right)\right\|}^{2}$$

To solve Equation (7), the centroid of each point set is first calculated as:

(8) $${\bar{P}}_{RCS}=\frac{1}{N}\sum_{i=1}^{N}p_{RCSi},\;{\bar{P}}_{OSI}=\frac{1}{N}\sum_{i=1}^{N}p_{OSIi}$$

where N=10 in this experiment. The point sets are then centralized by subtracting their respective centroids:

(9) $$P_{RCS}^{\prime }=P_{RCSi}-{\bar{P}}_{RCS},\;P_{OSI}^{\prime }=P_{OSIi}-{\bar{P}}_{OSI}$$

To solve for the optimal rigid-body transformation, a covariance matrix H is first constructed using the centralized point sets $P_{RCS}^{\prime }$ and $P_{OSI}^{\prime }$, as defined in Equation (10):

(10) $$H=\sum_{i=1}^{N}P_{RCS}^{\prime }{P_{OSI}^{\prime }}^{T}$$

The covariance matrix H encodes the correlation between the two point-sets in 3D space.

To extract the optimal rotation, Singular Value Decomposition (SVD) is applied to H, yielding:

(11) $$H=U\Sigma V^{T}$$

where U and V are orthogonal matrices containing the left and right singular vectors of H, respectively, and Σ is a diagonal matrix whose entries are the singular values. The optimal rotation matrix and the translation vector can be expressed as:

(12) $$R_{OSI}^{RCS}=VU^{T},\;t_{OSI}^{RCS}=-R_{OSI}^{RCS}{\bar{P}}_{OSI}+{\bar{P}}_{RCS}$$

#### 2.3.3. RCS-Sensor

The registration between the RCS and each individual sensor establishes the spatial relationship between the rigid coil structure and the OPM sensor array. Each excitation coil mounted on the RCS is modeled as an equivalent magnetic dipole with known position and magnetic moment in the RCS coordinate frame. During calibration, each coil is sequentially energized, and the magnetic field vectors are measured by all OPM sensors.

The magnetic field B(r) generated at a point by a steady current I flowing through a closed curve C is given by the Biot–Savart law:

(13) $$B(r)=\frac{\mu_{0}I}{4\pi }\oint_{C}\frac{dr^{\prime }\times \left(r-r^{\prime }\right)}{{\left|r-r^{\prime }\right|}^{3}}$$

where $\mu_{0}$ is the magnetic permeability of free space, $r^{\prime }$ denotes the position vector of the elemental line segment $dr^{\prime }$ on the current path c, and r is the observation point.

When the observation point is located sufficiently far from the current loop (distance much larger than the characteristic size of the coil), the loop can be approximated as a magnetic dipole with magnetic moment m. For a coil located at position $r_{c}$ with magnetic moment $m_{c}$, the theoretical magnetic field $B_{c,s}$ at the sensor position $r_{s}$ is described by the magnetic dipole equation:

(14) $$B_{c,s}=\frac{\mu_{0}}{4\pi }\left[\frac{3r_{c,s}(m_{c}\cdot r_{c,s})}{r_{c,s}^{5}}-\frac{m_{c}}{r_{c,s}^{3}}\right]$$

where $r_{c,s}=r_{c}-r_{s}$ is the position vector of the sensor with respect to the coil. The optimal position and orientation of each sensor in the RCS frame are obtained by minimizing the mean squared error between the measured and theoretical magnetic fields across all coils. Detailed derivations and experimental validation of this method are described in our previous work [34].

**Figure 5:**
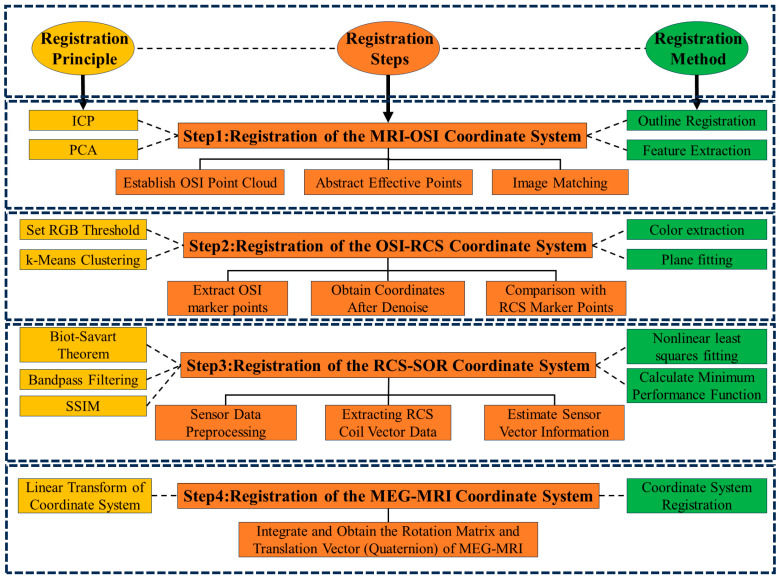
Overall workflow of the MEG–MRI co-registration framework.

The overall registration framework combining the MRI–OSI, OSI–RCS, and RCS–Sensor stages is illustrated in Figure 5.

A detailed mathematical description of all processing modules used in the workflow (including PCA, feature extraction, k-means clustering, plane fitting, SSIM-based similarity computation, and quaternion rotation representation) is provided in Appendix C.

## 3. Experiments and Results

Based on the Biot–Savart law (Equation (13)) and the magnetic dipole model (Equation (14)), a systematic simulation analysis was conducted to verify the applicability range of the magnetic dipole approximation. The simulations were designed to evaluate the equivalence accuracy of the magnetic dipole model under different coil and sensor configurations, including variations in coil dimensions, wire diameters, and sensor–coil distances.

The objective was to assess how these structural parameters affect the dipole approximation accuracy with respect to the true magnetic field generated by the current-carrying coil.

### 3.1. Simulation Model Construction

According to the experimental parameters described in reference [35], the excitation coil was modeled as a 40-turn cylindrical helix wound with 0.4 mm enameled copper wire, having an inner diameter of 4 mm. The distance between the coil center and the OPM sensor was varied from 80 mm to 250 mm during simulation. The coil model was constructed based on a standard cylindrical helical geometry, whose spatial equation $r_{c}^{h}$ is expressed as:

(15) $$r_{c}^{h}(x_{h},y_{h},z_{h})=\left\{\begin{array}{l} x_{h}=r_{h}\cos\alpha_{h}\\ y_{h}=r_{h}\sin\alpha_{h}\\ z_{h}=h_{h}*\alpha_{h}/2\pi \end{array}\right.\alpha_{h}\in [0,2\pi *n_{h}]$$

where $r_{h}$ denotes the radius of the helix, $h_{h}$ is the pitch of the helix (corresponding to the wire diameter including insulation thickness), $n_{h}$ is the total number of turns, the parameter $\alpha_{h}$ ranges from 0 to 2π, representing a complete winding of the helical coil.

Next, we will proceed with the modeling using the known conditions from the literature. The radius of the helix $r_{h}$ was set to 4 mm, the pitch of the helix $h_{h}$ was 0.4 mm, the total number of turns was 40. The distance between the coil center and the sensor $\left|r_{c,s}\right|$ ranged from 80 mm to 250 mm.

Each sensor measured only the magnetic field component along its sensitive axis, which corresponds to the single-axis output characteristic of the QuSpin Gen-II OPM sensor. From the set of direction vectors, 17 orientation vectors were randomly selected, forming a 17 × 3 matrix ${\vec{r}}_{s}^{sim}=\left[r_{sx}^{sim},r_{sy}^{sim},r_{sz}^{sim}\right]$. Since the magnitude of the excitation current $I_{sim}$ does not affect the relative field distribution. By substituting the sensor matrix $r_{s}^{sim}$ and the helical coil geometry $n_{s}^{sim}$ into Equations (13) and (14), the magnetic field $B_{h}^{sim}\left(r\right)$ generated by the helical coil at each sensor position was computed to evaluate the dipole fitting consistency. The computation process is expressed as:

(16) $$B_{h}^{sim}\left(r\right)=\frac{\mu_{0}I_{sim}}{4\pi }\oint_{r_{c}^{h}}\frac{dr_{c}^{h}\times \left(r_{s}^{sim}-r_{c}^{h}\right)}{{\left|r_{s}^{sim}-r_{c}^{h}\right|}^{3}}$$

For each observation point, the three orthogonal components of the magnetic field were integrated separately, yielding Equation (17):

(17) $$\begin{array}{l} B_{hx}^{sim}=\frac{\mu_{0}I}{4\pi }\int_{0}^{2\pi *n_{h}}\frac{r_{h}\cos\alpha_{h}(z_{s}^{sim}-z_{h})-2\pi h_{h}(y_{s}^{sim}-y_{h})}{\left[x_{s}^{sim}-x_{h},y_{s}^{sim}-y_{h},z_{s}^{sim}-z_{h}\right]}d\alpha_{h}\\ B_{hy}^{sim}=\frac{\mu_{0}I}{4\pi }\int_{0}^{2\pi *n_{h}}\frac{2\pi h_{h}(x_{s}^{sim}-x_{h})+r_{h}\sin\alpha_{h}(z_{s}^{sim}-z_{h})}{\left[x_{s}^{sim}-x_{h},y_{s}^{sim}-y_{h},z_{s}^{sim}-z_{h}\right]}d\alpha_{h}\\ B_{hz}^{sim}=\frac{\mu_{0}I}{4\pi }\int_{0}^{2\pi *n_{h}}\frac{-r_{h}\sin\alpha_{h}\left[\left(y_{s}^{sim}-y_{h})-(x_{s}^{sim}-x_{h}\right)\right]}{\left[x_{s}^{sim}-x_{h},y_{s}^{sim}-y_{h},z_{s}^{sim}-z_{h}\right]}d\alpha_{h} \end{array}$$

The final magnetic field simulation results formed a 50 × 17 matrix $B_{h}^{sim}$, containing a total of 850 scalar magnetic field values that represent the magnetic field intensities measured at 50 sensor locations along 17 orientation directions.

The corresponding simulated magnetic field $B_{d}^{sim}$ using the magnetic dipole approximation was calculated according to Equation (14), as expressed in Equation (18):

(18) $$B_{d}^{sim}=\frac{\mu_{0}}{4\pi }(\frac{3r_{c,s}^{sim}(m_{d}^{sim}\cdot r_{c,s}^{sim})}{{\left|r_{c,s}^{sim}\right|}^{5}}-\frac{m_{d}^{sim}}{{\left|r_{c,s}^{sim}\right|}^{3}}){\vec{r}}_{s}^{sim}$$

Here, $m_{d}^{sim}$ denotes the magnetic moment of the simulated dipole, and $r_{c,s}^{sim}=r_{d}^{sim}-r_{s}^{sim}$ represents the position vector from the coil center to the sensor. The magnetic dipole is equivalent to a circular loop carrying an electric current in space.

In the simulation, the helical coil was modeled as a circular loop, and the overall magnetic field was calculated by two approaches:

1. treating the entire helix as a single equivalent magnetic dipole;
2. summing the magnetic dipole contributions from each individual turn.

The difference between the two methods was found to be negligible.

The coordinate of the equivalent dipole point was set as $r_{d}^{sim}=[0,0,\frac{h_{h}*n_{h}}{2}]$, and the direction of the magnetic moment was defined as ${\vec{r}}_{d}^{sim}=[0,0,1]$. The magnetic moment magnitude was determined by the product of the coil current and the loop area $S_{d}$. Thus, the magnetic field of the dipole can be expressed as:

(19) $$B_{d}^{sim}=n_{h}\cdot \frac{\mu_{0}}{4\pi }\left\{\frac{3\left(r_{d}^{sim}-r_{s}^{sim}\right)\left[I_{sim}\cdot S_{d}\cdot {\vec{r}}_{d}^{sim}\cdot \left(r_{d}^{sim}-r_{s}^{sim}\right)\right]}{{\left|\left(r_{d}^{sim}-r_{s}^{sim}\right)\right|}^{5}}-\frac{I_{sim}\cdot S_{d}\cdot {\vec{r}}_{d}^{sim}}{{\left|\left(r_{d}^{sim}-r_{s}^{sim}\right)\right|}^{3}}\right\}{\vec{r}}_{s}^{sim}$$

According to Equation (19), the magnetic field generated by the equivalent magnetic dipole under the Biot–Savart condition was obtained as $B_{h}^{sim}$.

**Figure A1:**
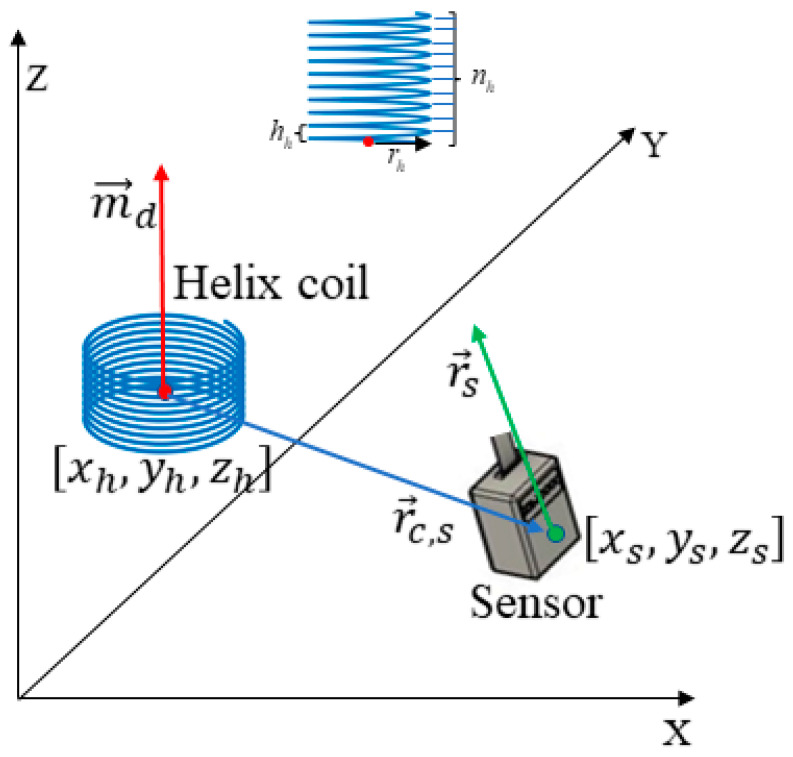
Geometric schematic and parametrization of the cylindrical helical excitation coil.

A complete schematic of the helical coil geometry, model parameters, and coil–sensor spatial relations is provided in Appendix B Figure A1.

We computed the ratio between the magnetic fields obtained from the Biot–Savart integration and from the dipole approximation, and used this ratio to evaluate the approximation under the conditions reported in reference [34]. Specifically, the relative dipole-equivalence fitting error $E=\left|B_{h}^{sim}/B_{d}^{sim}-1\right|$ was defined to quantify the degree of approximation. The statistical distribution of the simulation errors is presented in Table 1.

Table 1 shows that fewer than 50% of the sampled points have an error below 0.30, indicating that under the specified parameter settings the dipole approximation yields relatively low accuracy. Significant deviations are observed at certain sensor locations, which indicates that within the 80–250 mm distance range the dipole model is not uniformly valid for all positions.

### 3.2. Simulation of Coil Parameter Optimization

To determine the optimal coil configuration for subsequent system simulations, a parameter screening analysis was performed. The purpose of this step was to identify coil parameters that achieve a balance between magnetic dipole equivalence accuracy and engineering feasibility. Based on the verification results in the previous section, the coil geometry was further analyzed by varying its key structural parameters, including wire diameter, inner diameter, and number of turns. Other conditions, such as the excitation current and sensor configuration, were kept consistent with the settings described in reference [34]. This parameter optimization process provided guidance for selecting appropriate coil dimensions in the following simulations.

**Figure 6:**
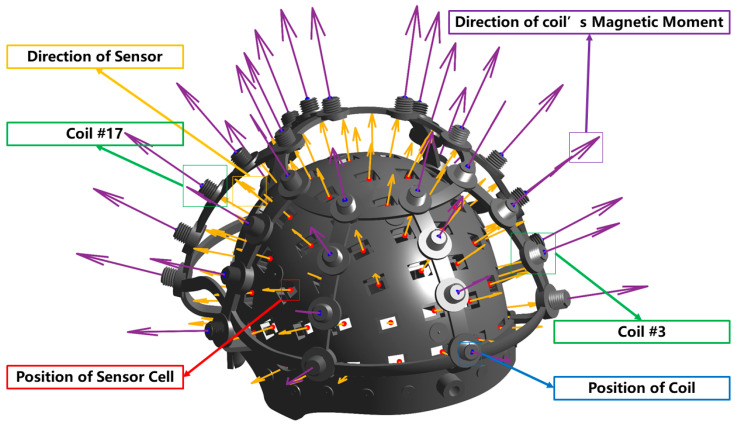
Simulation and experimental setup of the RCS–helmet model.

To validate the magnetic dipole equivalence under realistic system geometry, the rigid coil structure (RCS) developed in this study was utilized. Two representative coils (Coil #3 and Coil #17 in Figure 6) were selected from the RCS, and for each coil, five spatially distinct correspondence points on the sensor array were identified, resulting in a total of 10 coil–sensor pairs for analysis. This selection ensures that the analysis is not restricted to a single local region and reflects performance across different coil placements. Each pair represents one specific geometric configuration between a coil and a sensor, and the entire dataset captures the spatial variability inherent in the RCS layout.

In the following parameter study, three coil geometry parameters—inner diameter, wire diameter, and number of turns—were systematically varied, while other experimental conditions were kept constant. For each parameter combination, the magnetic field at the 10 sensor locations was computed using both the Biot–Savart integral (Equation (13)) and the magnetic dipole model (Equation (14)). The dipole-equivalence fitting error E was calculated for each coil–sensor pair, quantifying the local approximation accuracy. Statistical aggregation of these 10 pairwise errors was then used to evaluate how different coil geometries affect the validity of the dipole approximation within the actual RCS configuration.

Since the objective of this analysis was to evaluate parameter sensitivity rather than spatial distribution, it was sufficient to keep the coil–sensor positions fixed and control all other variables. Therefore, exhaustive sampling of all spatial locations was unnecessary. This design ensured fair comparison across different coil parameters while maintaining consistent geometric and positional conditions.

This RCS-based validation method preserves the geometric realism of the real system, while maintaining computational tractability through a limited yet representative sample set. The results obtained from this simulation serve as the basis for identifying coil parameter ranges that balance approximation accuracy and practical manufacturability. The spatial arrangement of the RCS coils and corresponding sensor locations is illustrated in Figure 6.

Figure 6—Illustration of the rigid coil structure (RCS), showing the spatial arrangement of all excitation coils and their magnetic moment directions (purple vectors), along with the sensor cell positions (red markers) and sensor orientation directions (yellow vectors). The figure visualizes the geometric relationship between the coils and OPM sensors used for dipole–field mapping and localization.

#### 3.2.1. Simulation of Wire Diameter Variation

To investigate the influence of wire diameter on the accuracy of the magnetic dipole approximation, a series of simulations were conducted under a fixed inner diameter $r_{h}$ was 4 mm and a fixed number $n_{h}$ was 10 turns. The wire diameter $h_{h}$ was set to 0.1 mm, 0.3 mm, and 0.5 mm, representing three typical fabrication configurations. All other experimental and geometric parameters remained constant.

Table 2 presents the dipole-equivalence fitting errors E of two representative coils (Coil 1 and Coil 2) under three wire diameters of 0.1 mm, 0.3 mm, and 0.5 mm. Each coil was paired with five sensor positions to evaluate the deviation between the Biot–Savart integral and the magnetic dipole model.

The results clearly show that the fitting error monotonically increases with wire diameter, indicating that thinner wires yield more accurate dipole-equivalent behavior. This trend arises because, for a cylindrical helical coil, a smaller wire diameter better approximates an ideal line current distribution, reducing geometric self-spreading of the magnetic field and improving the local uniformity. When the wire diameter increased from 0.1 mm to 0.5 mm, the average error approximately quadrupled, confirming that the magnetic dipole approximation becomes less valid as the coil thickness increases.

Therefore, under the assumption of a cylindrical helical coil, a smaller wire diameter consistently leads to higher dipole-approximation accuracy, and the 0.1 mm configuration provides the best fidelity among the tested cases.

#### 3.2.2. Simulation of Number of Turns Variation

To investigate the influence of the number of turns $n_{h}$ on the magnetic dipole approximation accuracy, a series of simulations were performed while keeping all other parameters constant. The inner diameter of the coil $r_{h}$ was fixed at 4 mm, and the wire diameter $h_{h}$ at 0.5 mm. Since the 0.1 mm wire diameter yields extremely high approximation accuracy with very small errors, it was less suitable for distinguishing the effect of coil turns. Therefore, a wire diameter of 0.5 mm was selected to make the variation in fitting error more visually and quantitatively comparable. The coil–sensor spatial configuration remained the same as that defined in the previous subsection, ensuring positional consistency for all tests.

Three representative turn numbers—10 turns, 20 turns, and 40 turns—were selected for comparison. Each coil model was constructed as a standard cylindrical helical winding, following the same pitch and geometric definition as in Equation (16). For each configuration, the magnetic field at 10 predefined coil–sensor pair was calculated using both the Biot–Savart integral (Equation (13)) and the magnetic dipole model (Equation (14)).

The relative deviation between the two results was quantified as the fitting error E, which reflects the dipole-equivalence accuracy under different coil turn numbers.

Table 3 summarizes the magnetic dipole fitting errors E of two representative coils (Coil 1 and Coil 2) under three different turn numbers: 10, 20, and 40. Each value represents the average error calculated at five coil–sensor pairs per coil based on the Biot–Savart and dipole-model field comparison.

The results show a slight increase in fitting error with the number of turns, indicating that adding more turns does not necessarily improve the dipole-equivalence accuracy.

This trend can be attributed to the extended spatial distribution of current in multi-turn windings, which deviates further from the point-dipole assumption.

However, this effect remains relatively small compared with the influence of wire diameter. In practical systems, increasing the number of turns also enhances the coil’s output magnetic moment and thereby improves the signal-to-noise ratio (SNR) in sensor measurements.

Therefore, the optimal turn number should be determined by balancing modeling precision and SNR performance rather than minimizing the dipole-fitting error alone.

#### 3.2.3. Simulation of Inner Diameter Variation

To evaluate the effect of coil size on the magnetic dipole approximation, a series of simulations $n_{h}$ were conducted with 10 turns and a wire diameter $h_{h}$ was set to 0.5 mm, while varying the inner diameter of the coil $r_{h}$ among 4 mm, 6 mm, and 8 mm. All other experimental conditions, including the coil–sensor spatial configuration, remained identical to those in the previous subsection.

For each configuration, the magnetic field at the 10 predefined coil–sensor pair was calculated using the Biot–Savart integral (Equation (13)) and the magnetic dipole model (Equation (14)). The relative deviation between the two results was quantified as the dipole-equivalence fitting error E.

The results of Table 4 reveal a clear decreasing trend of fitting error with increasing coil inner diameter. As the coil becomes larger in diameter and flatter in shape, the distribution of the magnetic field becomes more uniform, and the dipole approximation achieves higher fidelity. This effect is particularly evident when comparing the 4 mm and 8 mm configurations, where the average fitting error decreases by more than 70%.

Overall, for cylindrical helical coils, a flatter coil geometry (larger diameter relative to its axial length) provides better magnetic dipole equivalence. This indicates that the planarized or disk-like winding structures are more suitable for dipole-based modeling in RCS applications, as they exhibit field characteristics closer to an ideal magnetic dipole source.

The preceding simulations focused on cylindrical helical coils, which are widely used due to their ease of fabrication and compatibility with dense sensor arrays. However, this model inherently assumes a finite axial length, and the resulting magnetic field distribution may deviate from the ideal point-dipole pattern, especially when the coil pitch or height is not negligible compared to its radius.

To address this limitation, another typical configuration—the circular loop (single-turn) coil—is considered here. This geometry represents the limiting case of the helical structure when the winding pitch tends to zero and only one turn remains. In this case, the coil can be idealized as a flat circular ring, whose magnetic field characteristics are expected to better conform to the dipole approximation due to its planar symmetry.

The spatial equation of the circular loop lying in the *x*–*y* plane and centered at the origin is defined as Equation (20):

(20) $$r_{c}^{l}(x_{l},y_{l},z_{l})=\left\{\begin{array}{l} x_{l}=r_{l}\cos\alpha_{l}\\ y_{l}=r_{l}\sin\alpha_{l}\\ z_{l}=0 \end{array}\right.,\alpha_{l}\in [0,2\pi ]$$

where $r_{l}$ denotes the radius of the loop, the parameter $\alpha_{l}$ ranges from 0 to 2π, representing a circle of the loop.

For the circular loop coil, the magnetic field computation follows the same procedure as described in the cylindrical helical coil simulation, but with a simplified geometry since there is only one turn and no axial extension. Both the Biot–Savart integration and magnetic dipole fitting processes were performed in the same numerical framework. Because the analytical form of the circular loop is straightforward, the detailed derivation is omitted here.

In this simulation, the coil radius was set to 4 mm, 6 mm, and 8 mm, corresponding to the same spatial configuration as the helical coil experiments. The coil is assumed to be a single-turn loop without wire thickness or inter-turn spacing, and all other parameters, including the sensor positions and orientations, were kept identical to ensure direct comparability. The fitting error E was calculated by comparing the magnetic field results obtained from the Biot–Savart integral and the magnetic dipole model under the same conditions.

Table 5 shows very small dipole-fitting errors for the single-turn circular loop cases (errors on the order of 10^−3^), and a clear trend: the smaller the loop radius, the smaller the fitting error. Two main physical reasons explain this behavior:

- Geometric point-like approximation.

A magnetic dipole is the far-field equivalent of a spatially compact current loop. When the loop radius rrr is much smaller than the sensor distance $\left|r_{c,s}^{sim}\right|$, the actual field produced by the loop approaches the ideal dipole field and higher-order multipole terms become negligible. Reducing $r_{l}$ therefore decreases the relative contribution of non-dipolar components, producing a lower fitting error.

- Planar symmetry and reduced axial extent.

A single circular loop geometrically planar and compact: its current distribution is concentrated in a small, nearly planar ring. Such planar symmetry produces a field whose angular dependence and radial decay match the dipole pattern closely in the measurement region. Consequently, smaller-radius rings—being more compact—match the dipole model better.

Why this differs from cylindrical helical coils:

- Axial extension and distributed current: a cylindrical helical coil current distributed over multiple turns and along an axial length. That extended 3-D current distribution introduces significant non-dipolar (higher-order multipole) contributions in the near and intermediate fields, increasing the dipole-fitting error compared with a planar single-turn loop of the same outer radius.
- Effective aspect ratio (axial length vs. radius): helical coils with appreciable axial height are less “point-like” even if their outer radius is similar. In contrast, a flattened (disk-like) coil or single loop minimizes axial extent and so is closer to an ideal dipole.

For RCS designs where representing coils as magnetic dipoles is desired, planar (flattened) loop geometries—and smaller radii in particular—provide the best dipole equivalence. However, this must be balanced with engineering trade-offs: very small loops reduce magnetic moment and SNR, while flattened single-turn loops may be less practical for generating required field amplitude than multi-turn helices. Thus, coil geometry selection should balance dipole-model fidelity and practical SNR/engineering constraints.

### 3.3. Experimental Validation of the RCS-Based Sensor Localization System

#### 3.3.1. Coil Localization Experiment

The experimental validation was conducted using the rigid coil structure (RCS) designed in this study, which provides fixed and precisely known coil positions for accurate reference measurements. The RCS consists of two integrated coil units rigidly attached to a 3D-printed base frame, each containing five color-marked sensors corresponding to ten coil–sensor reference pairs. This configuration reproduces the spatial relationships of a flexible OPM-MEG cap while maintaining mechanical stability during measurement.

Each coil was fabricated following the optimized parameters determined from the previous simulations, and the magnetic dipole fitting reliability of the RCS configuration has already been experimentally verified in Ref. [34]. Therefore, the present experiment focuses on evaluating the practical co-registration accuracy of the complete localization workflow, rather than revalidating the dipole equivalence model itself.

The coil current driving and signal acquisition system was identical to that used in Section 2.2, consisting of a Keysight 33500B function generator and a Stanford CS580 current source. The function generator produced a sinusoidal voltage waveform that served as the input for the current source, which in turn supplied stable AC current to each coil sequentially. The driving frequency was selected according to the local electromagnetic noise spectrum to avoid overlap with line-frequency interference. During each measurement cycle, the induced magnetic field signals were collected by the OPM sensors placed at the predefined RCS locations and stored for subsequent co-registration analysis.

**Figure 7:**
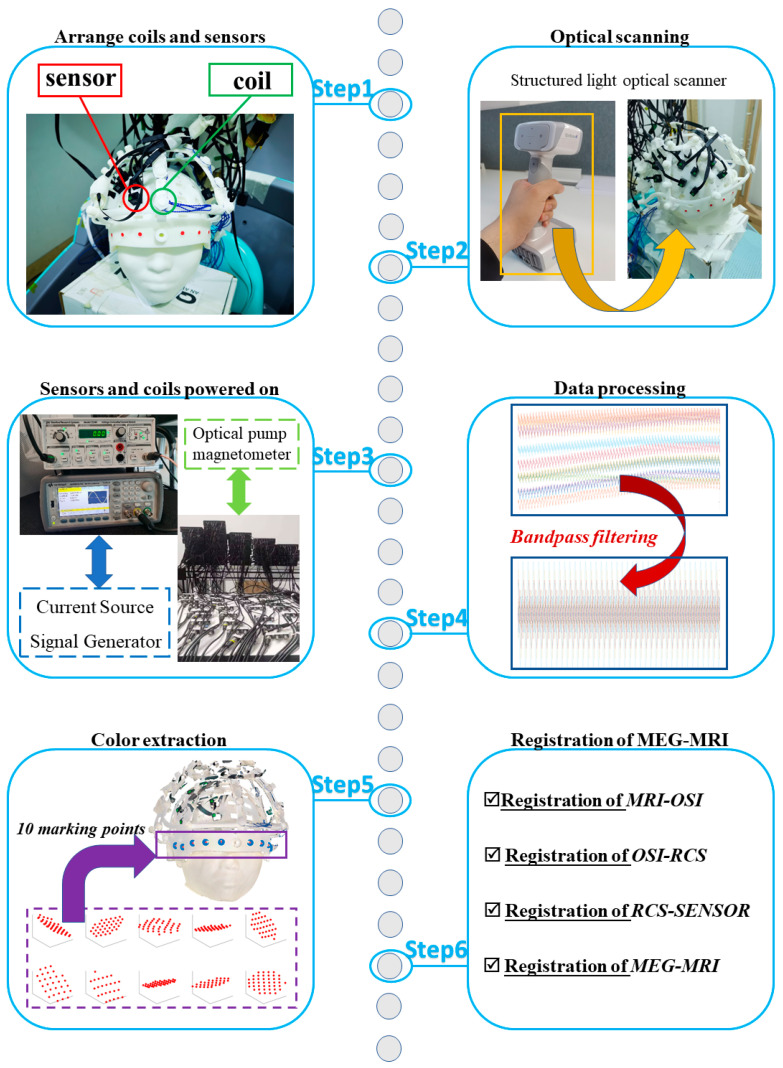
Overall experimental flowchart.

The experimental workflow followed six sequential steps, as illustrated in Figure 7, to complete the full co-registration process from magnetic field acquisition to MEG–MRI alignment.

The rigid coil structure (RCS) and helmet frame were first fixed on the head model, and all OPM sensors and excitation coils were installed at their designated locations. Each excitation coil was a single-turn circular loop with an inner diameter of 6 mm, fabricated using enameled copper wire. This configuration was selected based on previous simulations, as a single-turn coil minimizes model uncertainty and mechanical deformation while maintaining sufficient magnetic moment for stable measurements. The helmet ID and coil numbering were recorded for reference.

The number and placement of the coils in the RCS were based on the 85-channel rigid OPM helmet developed in our laboratory (see Ref. [10]). The coil layout follows the spatial arrangement of the helmet’s sensor array, considering head curvature, inter-sensor spacing, and the overall geometric constraints of the rigid structure. The feasibility and usability of this coil–sensor configuration have been previously validated in Ref. [34].

All OPM sensors and excitation coils were installed at their designated locations on the rigid helmet. The helmet was manufactured using high-precision 3D printing, achieving a dimensional tolerance better than 0.1 mm, which ensures highly accurate sensor positioning. The excitation coils were wound and mounted manually, resulting in an additional positional uncertainty within 0.3 mm and an orientation deviation typically below 0.3–0.5°. Moreover, Ref. [34] has evaluated the robustness of magnetic dipole fitting under perturbed coil-assembly errors (random positional perturbations up to 0.5 mm and angular perturbations up to 0.5°), confirming that the approach remains fully effective under such conditions.

Step 1: Arrangement of coils and sensors

The rigid coil structure (RCS) and helmet frame were fixed on the head model. All OPM sensors and excitation coils were installed at their predefined positions, and both the helmet ID and coil numbering were recorded for reference.

Step 2: Optical scanning

A structured-light optical scanner was used to capture the 3D surface geometry of the helmet markers and facial contour. The scanning process generated an optical surface image (OSI) file that served as the geometric reference for subsequent registration.

Step 3: Powering the sensors and coils. The OPM sensors were first powered on to measure the background magnetic field. Then, each excitation coil was sequentially driven by a function generator and a current source, and the magnetic field responses recorded by the sensors were stored for later processing.

Step 4: Data processing

The sensor outputs were time-aligned and filtered to remove environmental noise, mainly using band-pass filtering to isolate the coil-induced magnetic field components.

Step 5: Color extraction

RGB threshold segmentation was applied to extract the color-coded fiducial markers from the OSI, thereby determining the spatial coordinates of the ten marker points on the RCS.

Step 6: MEG–MRI registration

The complete registration pipeline was executed, including the MRI–OSI, OSI–RCS, RCS–Sensor, and final MEG–MRI co-registration stages, to obtain the unified spatial transformation linking the MRI anatomical space and the MEG sensor coordinate system.

#### 3.3.2. Experimental Results and Analysis

A total of 18 excitation coils and 12 OPM-sensors were arranged on the rigid coil structure (RCS) platform for the experimental validation. However, one sensor exhibited abnormal noise and instability during the measurement, and its data were excluded from analysis. Therefore, the final dataset included 11 valid sensors, corresponding to 198 coil–sensor measurement pairs in total.

Although the RCS provides 35 possible coil positions, only 18 coils were activated in this experiment because this subset already provides sufficient spatial excitation for the sensors in our test configuration. Using a reduced number of coils does not affect the validity of the proposed method, as the optimization relies only on the coils that are actually driven.

Single-turn circular coils were chosen because their minimal axial thickness and uniform current distribution produce magnetic fields that closely follow an ideal dipole model at practical sensor distances. In contrast, multi-turn coils introduce axial extension and additional geometric variability, which increase dipole-approximation error. Therefore, single-turn coils offer higher model fidelity for dipole-based localization.

Note that the QZFM OPM sensors used in this study are specified with a dynamic range of ±5 nT. Under our coil excitation condition (1 mA) and the present helmet/coil geometry, Biot–Savart–based numerical calculations indicate that the maximum magnetic field at the nearest sensor remains below ~0.2 nT (200 pT). This value is more than one order of magnitude lower than the sensor limit, providing a substantial safety margin and ensuring that all sensors operate well within their linear response region. No saturation or non-linear distortion is expected under these conditions.

The experimental procedure followed the six-step workflow shown in Figure 7. Each coil was sequentially energized, and the magnetic field responses measured by all active sensors were recorded. The magnetic dipole fitting method described in Section 2.3 was then applied to determine each coil’s estimated position and orientation. The fitted results were compared with their ground-truth positions measured from the OSI to evaluate the co-registration accuracy.

The localization performance was quantified using two metrics the position error, defined as the Euclidean distance between the fitted and reference coil centers, and the orientation error, defined as the angular deviation between the estimated and true dipole moment directions.

As summarized in Table 6, the RCS-based co-registration experiment yielded an average position error of 4.80 mm and an average orientation error of 5.27° across 11 valid OPM sensors. The minimum error reached 2.12 mm/3.35°, while the maximum deviation was 9.41 mm/10.09°. These results demonstrate that the experimental system achieved a few millimeters localization accuracy under physical measurement conditions.

To further refine the spatial alignment precision, an optical correction method was subsequently introduced. This approach employs high-resolution geometric data from optical scanning to perform fine alignment corrections after magnetic dipole fitting. The corrected registration results are presented in the following section.

#### 3.3.3. Optical Constraint–Assisted Sensor Orientation Correction

In this section, the optical correction was conducted as part of the same experiment described above. The correction aims to determine the approximate orientation of each sensor’s sensitive axis by scanning the color markers on the top surface of the OPM sensor modules. As shown in Table 6, although most sensors in the pure-coil localization experiment achieved millimeter-level spatial accuracy, several channels still exhibited orientation estimation deviations and local convergence instability. These errors mainly arise from the high degree of freedom in the sensitive-axis orientation when relying solely on the magnetic dipole model. Under conditions of local magnetic field asymmetry or low signal-to-noise ratio, the optimization may suffer from multiple local minima or axis reversal issues.

To obtain the surface normal of each sensor housing, we first apply a color-based threshold to the structured-light point cloud to isolate the colored markers printed on the top of each housing. The extracted marker points are then fitted with a plane using a least-squares method, and the resulting plane normal is used as the optical normal vector.

To address this, the same set of experimental data was reprocessed by incorporating directional constraint information obtained from optical scanning to assist and refine the sensor orientation estimation. In the experimental setup, optical scanning served not only to achieve global registration between the OSI and RCS coordinate systems, but also to provide an approximate reference for the sensitive-axis orientation of each sensor. Specifically, structured-light scanning was used to capture the 3D coordinates of external RCS markers and the color marker points on each sensor housing. For each sensor, the color marker points extracted from its top surface were fitted to a plane, from which the approximate surface normal vector was calculated. This normal vector was regarded as the initial estimation of the sensor’s sensitive-axis orientation and was then expressed in spherical coordinates, where the polar angle is denoted by $\theta_{p}$ and the azimuthal angle by $\phi_{p}$.

To prevent over-constraining the optimization space while suppressing instability, relative angular constraints were applied to the spherical coordinate parameters. The sensitive-axis direction vector was represented in spherical coordinates as ${\hat{m}}_{c}=[\theta_{c},\phi_{c},1]$, and two constraint ranges were adopted in the localization experiments:

-
$\theta_{c}\in [\theta_{p}(1-0.05),\theta_{p}(1+0.05)],\;\phi_{c}\in [\phi_{p}(1-0.05),\phi_{p}(1+0.05)];$

-
$\theta_{c}\in [\theta_{p}(1-0.01),\theta_{p}(1+0.01)],\;\phi_{c}\in [\phi_{p}(1-0.01),\phi_{p}(1+0.01)];$

During optimization, the optical constraint information was embedded into the search process of the objective function as the boundary condition for the sensor orientation.

Apart from this additional constraint, the overall algorithmic framework remained identical to that used in the pure-coil localization experiment, employing a magnetic dipole–based multi-start constrained optimization method (MultiStart + fmincon) for solution estimation.

The angular limits of ±5% and ±1% were selected based on the measured orientation accuracy of the structured-light scanner (approximately 0.5–1.2°) and preliminary robustness tests. The ±5% range corresponds to the typical deviation of the optical normal relative to the nominal sensor tilt, whereas the ±1% bound represents a more stringent constraint used to assess the benefit of tighter optical guidance. Additional simulations with constraint ranges between ±0.5% and ±10% showed comparable accuracy, although excessively tight bounds (≤±0.5%) may lead to over-constraining and slower convergence.

The ±5% constraint corresponds to the typical variability of the optical normals extracted across sensors and reflects reasonable manufacturing deviations of the housing surface. In contrast, the ±1% constraint represents a deliberately tighter bound, introduced to evaluate the influence of more stringent orientation guidance on the optimization process. These two ranges therefore correspond to a realistic tolerance and a strict-constraint scenario, respectively.

**Figure 8:**
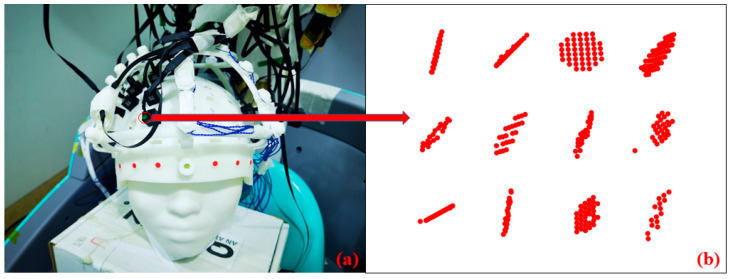
Optical extraction of color markers on sensor surfaces and corresponding fitted normal vectors: (**a**) Experimental setup; (**b**) Extracted color-marker point clouds for each sensor.

The extraction of the top-surface color markers and the fitted normal vectors for each sensor are shown in Figure 8, where panel (a) illustrates the experimental setup and panel (b) presents the reconstructed point clouds of the color markers.

Figure 8 illustrates the optical marker extraction procedure and the resulting point clouds used for sensor top-surface plane fitting. Panel (a) is a photograph of the experimental helmet with the rigid coil structure (RCS) and OPM sensor modules; the red circle highlights one sensor whose top-surface markers are shown in the corresponding subplot in (b). Panel (b) presents the digitized color-marker point clouds for multiple sensors: each small subplot contains the raw 3D coordinates of the extracted color-marker points from the top surface of one sensor, expressed in the structured-light scanner reference frame (axes in millimeters, mm). These red scatter points were fitted to a plane for each sensor; the resulting plane normal vectors were then used as optical orientation priors (soft constraints) in the dipole-based localization optimization. The plots in (b) therefore demonstrate both that reliable marker point clouds can be extracted for most sensors despite partial occlusion and surface variations, and the extracted clouds are sufficiently planar to yield meaningful orientation estimates for use as optical priors. For visualization clarity, panel (b) shows the raw marker clouds (red points); the fitted planes and normal vectors are reported numerically in the text and were used in the constrained optimization described in Section 3.3.3.

The sensor orientation localization results after applying optical constraints with ±5% and ±1% angular limits are summarized in Table 7.

The optical constraint calibration produced a substantial improvement in the overall localization accuracy, as shown in Table 7. Only the first four sensors exhibited errors identical to those obtained prior to optical correction, indicating that these sensors had already achieved physically reasonable orientation estimates during the initial magnetic fitting. For the remaining sensors, the introduction of optical angular constraints led to significant reductions in localization error, particularly under the tighter ±1% condition. This demonstrates that the optical surface-normal information provides effective guidance for stabilizing the optimization and correcting orientation deviations that occur in unconstrained dipole-fitting.

A small number of channels showed slightly better performance under the ±5% constraint than under ±1%. This phenomenon suggests that the true sensitive-axis orientations of those sensors may lie just outside the narrow ±1% interval, given that the optical plane-fitting procedure provides only an approximate estimation of the sensor top-surface normal rather than its exact physical sensitive-axis direction.

Overall, the results confirm that optical calibration plays a critical role in enhancing the robustness and accuracy of sensor orientation estimation, serving as an effective auxiliary constraint that greatly improves the stability and consistency of the magnetic-dipole–based localization process.

## 4. Discussion

### 4.1. Hardware Limitations and Their Impact on Coil-Based Localization Accuracy

Although the proposed RCS-based localization framework demonstrated a few millimeters accuracy performance in real measurements, the experimental accuracy is fundamentally constrained by the hardware limitations of the system. Among all hardware-related factors, the quality and physical integrity of the coils have the greatest impact on the localization results. In this study, the coils were fabricated manually, and while their overall precision is sufficient for typical MEG co-registration tasks, the results do not represent the upper limit of coil-based localization accuracy.

The dominant source of hardware error arises from the geometrical deviation between the physical coil and the ideal mathematical model. Even small structural imperfections—such as non-uniform winding tension, asymmetric deformation, or slight variations in the inner radius—directly alter the spatial current distribution and cause measurable discrepancies from the theoretical magnetic dipole field. In addition, the magnetic contribution of the wire terminals inevitably leads to partial field cancellation and introduces residual magnetization, both of which reduce the effective signal-to-noise ratio and degrade dipole-fitting accuracy. These effects cannot be fully eliminated by software post-processing and inherently limit the achievable accuracy of any coil-based localization method.

Other hardware imperfections also exist but contribute far less to the overall error. For example, the intrinsic precision of the OPM instruments and the 3D-printing tolerance of the RCS structure introduce minor geometric deviations, yet their magnitude is significantly smaller compared to coil-related errors. These secondary factors remain within a range that produces only negligible influence on the final co-registration outcome.

### 4.2. Software and Data-Collection Limitations

Beyond hardware factors, the localization accuracy is also influenced by software-level and data-collection uncertainties, particularly those arising from the process of aggregating measurements across multiple coil activations. During data acquisition, each coil is energized sequentially, and the magnetic field is recorded simultaneously by all sensors. Within a single coil activation, the timing across sensors is highly synchronized and does not introduce phase or latency errors.

Hardware-related manufacturing errors and their amplification in localization. While we verified the rigidity and gross geometry of the RCS in front-end tests (no evidence of structural deformation or mounting failure), our post-hoc analysis indicates that a dominant contributor to the larger localization errors observed for certain sensors is variability in the excitation coils arising from manual fabrication. Hand-wound multi-turn coils can exhibit non-negligible variations in effective radius, pitch, turn spacing, and wiring path; subtle differences in connector contacts or coil resistance may also occur. These deviations produce a mismatch between the real coil field and the idealized model (Biot–Savart/dipole) used in the inverse localization. Because the localization optimization is sensitive to model fidelity, such mismatches can be nonlinearly amplified—especially for sensors in peripheral helmet locations where field sensitivity is lower—resulting in the outlying errors reported in Table 6 and Table 7.

However, coil-based sensor localization requires aggregating the magnetic signatures produced by all coils for each individual sensor. This necessitates aligning multi-coil measurement batches that were acquired at different time points. Because each coil activation is triggered independently, small timing jitter—typically below the 1 ms sampling interval of the 1000 Hz OPM acquisition—unavoidably occurs between batches. These sub-millisecond offsets disrupt the precise temporal correspondence among the sensor signals across coils. Since each batch captures the sensor response at a slightly different effective sampling instant, the resulting misalignment can introduce small but non-negligible inconsistencies in the multi-coil data vector used for dipole fitting. This timing-alignment uncertainty constitutes an inherent software-level limitation of coil-based localization and partially explains the discrepancy observed between simulation and experimental results.

### 4.3. Limitations of the Magnetic Dipole Model

The magnetic dipole model serves as the theoretical foundation for coil-based sensor localization; however, its accuracy is inherently affected by several geometric and physical factors. As demonstrated in previous work [34], the three spatial angles between the coil and sensor, together with their separation distance, constitute the dominant determinants of dipole-model accuracy. Large relative angles or short sensor–coil distances cause the measured field to deviate from the ideal dipole pattern, leading to increased fitting error.

In addition to spatial configuration, the present study further demonstrates that the internal geometry of the coil has a substantial impact on dipole approximation accuracy. For cylindrical helical coils, the simulation results show that:

- Wire diameter is the most influential factor,
- Followed by the inner radius,
- While the number of turns also affects accuracy but to a lesser degree, since increasing the number of turns expands the spatial current distribution and weakens the point-like dipole assumption.

Moreover, coils with a flatter current distribution (i.e., pancake-type geometries) produce magnetic fields that more closely resemble an ideal dipole, consistent with the observed improvement in fitting accuracy for planar coil configurations.

For single-turn circular coils, the decisive parameter is the inner diameter: smaller radii yield stronger similarity to the dipole field and hence smaller approximation error. This follows directly from the fact that a smaller current loop more closely approaches a point magnetic dipole.

Nevertheless, whether using helical coils or single-turn coils, there exists an inherent trade-off between signal-to-noise ratio (SNR) and dipole-model accuracy. Smaller coils improve dipole approximation but simultaneously reduce magnetic field amplitude, which may compromise SNR and hinder reliable sensor localization. Therefore, practical coil design must balance these competing requirements to determine an optimal parameter range that maintains both adequate SNR and acceptable dipole-model accuracy.

### 4.4. Joint Analysis of the RCS Structure and Optical Orientation Constraints

The experimental results show that integrating the Rigid Coil Structure (RCS) with optical orientation constraints produces a complementary effect that enhances the robustness of the overall co-registration pipeline. The RCS provides a stable geometric framework that minimizes coil–sensor relative displacement and ensures consistent magnetic-field measurements, thereby supporting reliable dipole-based localization, as validated in our prior work [34].

The optical orientation constraint compensates for an inherent limitation of purely magnetic dipole optimization—namely, the weak observability of the sensor’s sensitive-axis orientation. When only magnetic measurements are used, the optimization may converge to local minima, exhibit axis-flip behavior, or become unstable, especially under unfavorable geometric conditions such as excessively large angles, excessively small angles, or near-orthogonal coil–sensor orientations. These configurations reduce magnetic-field asymmetry and make the orientation parameter difficult to resolve.

By extracting the planar normal vector from the RGB-based surface marker points, a coarse but physically reasonable initial direction can be obtained for each sensor. Restricting the polar and azimuth angles within ±5% or ±1% of this estimate limits the search domain to a plausible region. The experimental results (Table 7) reveal two important findings:

For a portion of the channels, the orientation results obtained with optical constraints remain consistent with those from unconstrained localization. This indicates that the RCS-based magnetic localization already places those sensors in a correct angular neighborhood, and the optical constraints mainly serve as a stability safeguard rather than altering the solution.

For another subset of channels, particularly those exhibiting larger orientation deviations in the unconstrained case, the optical constraints substantially improve direction accuracy. The ±1% range is the strictest but may exclude the true orientation if the extracted surface normal does not perfectly match the actual sensitive axis; in such cases, the ±5% range performs better.

Overall, combining RCS with optical orientation constraints stabilizes the optimization landscape, suppresses axis ambiguity, enhances directional accuracy under challenging coil–sensor geometries, and improves the consistency of multi-channel localization. Although coarse, the optical information provides a physically grounded boundary that magnetic data alone cannot guarantee.

### 4.5. Limitations and Role of the Magnetic-Field Relative Error E

Although the relative magnetic-field error E between the full Biot–Savart computation and the dipole-approximation model provides a convenient quantitative indicator of the approximation accuracy, it does not directly translate into the final localization accuracy in terms of position or orientation. This is because the sensitivity of the inverse localization process to magnetic-field perturbations is highly geometry-dependent, non-uniform across sensors, and jointly influenced by coil–sensor distances, field directionality, and conditioning of the optimization problem. Therefore, E should not be interpreted as a direct predictor of localization performance.

In this study, the primary purpose of using E is to guide the selection of coil geometry parameters and to verify that the RCS design operates within a regime where the dipole approximation remains sufficiently accurate for practical optimization. The simulation of E thus serves as an internal model-consistency check rather than a full predictor of experimental errors.

Nevertheless, we acknowledge that a more rigorous mapping between magnetic-domain approximation errors and the resulting spatial localization errors is desirable. Developing an effective evaluation framework that directly quantifies how dipole-model errors propagate to position/orientation deviations will be the focus of our future work. Such an analysis would enable a deeper understanding of approximation quality and could further optimize coil design and sensor placement in OPM-MEG systems.

### 4.6. Comprehensive Advantages, Applicability, and Future Directions of the Proposed Co-Registration Framework

While the experimental results demonstrate a positional accuracy on the order of a few millimeters, it is important to recognize that several unquantified hardware-related factors contribute to the reported error level. In the present study, the primary purpose of the coil modeling and sensor-localization framework is to establish a general methodology for dipole-based co-registration rather than to exhaustively characterize all hardware imperfections. Many hardware uncertainties—such as manufacturing deviations of the hand-wound helical coils, small variations in the sensor housing geometry, and residual optical-fitting errors—are difficult to isolate and quantify individually in the current prototype system. These factors can be amplified by the inversion process, especially for sensors located in geometrically unfavorable regions of the helmet, and likely constitute a non-negligible portion of the observed 5-mm average error.

Because of these challenges, the present paper does not attempt to provide a complete quantitative decomposition of all error sources. Instead, our focus is on demonstrating that the proposed RCS-based dipole localization architecture is feasible and robust under realistic experimental conditions. A systematic and quantitative evaluation of how individual error sources—such as coil geometry imperfections, modeling inaccuracies, residual optical-normal bias, and sensor noise—propagate into positional and orientational localization errors will be the focus of our future work. In particular, we plan to develop more refined simulation and calibration procedures to directly characterize the sensitivity of the localization results to specific hardware defects and dipole-approximation errors.

Although optical scanning–based localization can achieve very high geometric accuracy, it fundamentally operates on the outer housing of an OPM sensor rather than its internal sensitive point. In contrast, coil-based localization directly estimates the position of the sensor’s true sensing location, which is the coordinate relevant for MEG forward modeling. Even millimeter-level offsets between the housing and the vapor cell can lead to noticeable forward-modeling errors, making coil-based localization functionally irreplaceable. In addition, magnetic measurements are inherently insensitive to occlusion, visibility, or local lighting conditions, and remain robust under head motion—conditions where optical systems often degrade.

Meanwhile, optical information provides a valuable complementary benefit: it supplies stable orientation priors that mitigate the intrinsic ambiguity of dipole-based direction estimation. Importantly, in our framework optical data are collected offline during a controlled calibration step, when the RCS and markers are fully visible. The extracted surface normals are then used only as static prior constraints in the subsequent magnetic optimization. During actual MEG data acquisition and dynamic operation, the system depends solely on magnetic measurements, avoiding the runtime vulnerabilities of optical tracking such as occlusion, marker loss, and motion-induced instability. Thus, optical data enhance directional stability in the calibration/post-processing stage, while coil-based measurements ensure robustness during real-time operation—forming a genuinely complementary hybrid approach.

It should be emphasized that the key contribution of this study lies in its theoretical framework rather than its current experimental accuracy. The localization accuracy obtained in our experiments is limited by coil fabrication tolerance, wire-end residual magnetization, the geometric precision of the RCS, and scanner resolution, and therefore does not represent the upper limit of the proposed method. These practical limitations do not diminish the methodological value of our work. The dipole-model validity analysis, coil-geometry sensitivity evaluation, and hybrid optical–magnetic constraint formulation provide theoretical and practical guidance for future high-precision OPM-MEG systems.

Future improvements will be primarily hardware-driven. High-precision coil fabrication—such as PCB-based coils, automated precision winding, industrial-grade coil forming, and optimized wire-end treatments—may significantly reduce geometric deviations and residual magnetic artifacts, thereby improving dipole-model accuracy. Improved optical scanners, higher-resolution markers, and stiffer RCS structures may further reduce geometric uncertainty. As purely optical localization remains vulnerable to occlusion and marker degradation, the next generation of OPM-MEG systems is expected to rely increasingly on coil-based or hybrid optical–magnetic co-registration. Enhancing coil manufacturing precision will therefore be essential for pushing the performance boundary of future sensor-localization technologies.

## 5. Conclusions

In this study, we developed a comprehensive sensor localization and orientation framework for OPM-MEG systems based on rigid coil structures (RCS), magnetic dipole modeling, and optional optical orientation constraints. Through systematic simulation and experimental analyses, the proposed method establishes a unified theoretical foundation for achieving high-accuracy, high-robustness co-registration across different acquisition conditions.

First, we quantitatively evaluated the intrinsic limitations and applicability of the magnetic dipole approximation, revealing how coil–sensor spatial configuration and coil internal geometry jointly determine localization accuracy. These findings provide practical guidelines for coil design, parameter selection, and error control in future OPM-MEG systems. Second, we implemented a complete coil-based sensor localization workflow and validated it experimentally using an 18-coil, 11-sensor RCS setup. The results demonstrate that, even under non-ideal coil fabrication tolerances, the proposed scheme achieves millimeter-level positional accuracy. Third, we introduced optical surface-normal–based orientation priors as an auxiliary constraint. This hybrid strategy effectively stabilizes direction estimation for the minority of channels in which magnetic-only optimization exhibits local non-convergence or orientation ambiguity.

It is important to note that the experimental accuracy reported here does not represent the upper limit of the proposed framework. The observed errors are dominated by coil-manufacturing imperfections, residual magnetization, RCS fabrication tolerances, and scanning resolution, rather than limitations of the theoretical method itself. The methodological contributions—dipole-model characterization, coil-geometry sensitivity analysis, and hybrid magnetic–optical optimization—remain broadly applicable and provide actionable guidance for designing next-generation OPM-MEG co-registration systems.

Future improvements will focus on hardware refinement, such as PCB-based precision coils, automated winding, optimized wire-end treatment, and higher-resolution scanning. These enhancements are expected to further reduce systematic errors and push coil-based localization toward its theoretical performance limits. Overall, the proposed framework offers a principled, extensible pathway for accurate and robust sensor localization in emerging OPM-MEG technologies.

## Figures and Tables

**Table 1:** Simulation Error Statistics for Magnetic Dipole Equivalence Verification Based on the Parameter Conditions.

$\left|E\right|$	Number of Point	Proportion
<0.02	107	12.59%
<0.05	180	21.18%
<0.10	230	27.06%
<0.20	314	36.94%
<0.30	382	44.94%

**Table 2:** Simulation results of magnetic dipole fitting errors under different Wire diameters.

	*E* *h_h_* = 0.1 mm	*E* *h_h_* = 0.3 mm	*E* *h_h_* = 0.5 mm		*E* *h_h_* = 0.1 mm	*E* *h_h_* = 0.3 mm	*E* *h_h_* = 0.5 mm
Coil1	0.0085	0.0271	0.0458	Coil2	0.0018	0.0069	0.0120
	0.0040	0.0106	0.0171		0.0106	0.0304	0.0500
	0.0071	0.0231	0.0391		0.0022	0.0049	0.0075
	0.0098	0.0308	0.0519		0.0098	0.0307	0.0516
	0.0099	0.0312	0.0526		0.0160	0.0495	0.0831

**Table 3:** Simulation results of magnetic dipole fitting errors under different turns.

	*E* *n_h_* = 10	*E* *n_h_* = 20	*E* *n_h_* = 40		*E* *n_h_* = 10	*E* *n_h_* = 20	*E* *n_h_* = 40
Coil1	0.0458	0.0493	0.0541	Coil2	0.0120	0.0135	0.0173
	0.0171	0.0172	0.0174		0.0500	0.0521	0.0525
	0.0391	0.0423	0.0475		0.0075	0.0079	0.0086
	0.0519	0.0556	0.0604		0.0516	0.0553	0.0599
	0.0526	0.0564	0.0613		0.0831	0.0885	0.0942

**Table 4:** Simulation results of magnetic dipole fitting errors under different inner diameters.

	*E* *r_h_* = 4 mm	*E* *r_h_* = 6 mm	*E* *r_h_* = 8 mm		*E* *r_h_* = 4 mm	*E* *r_h_* = 6 mm	*E* *r_h_* = 8 mm
Coil1	0.0458	0.0199	0.0100	Coil2	0.0120	0.0049	0.0015
	0.0171	0.0073	0.0049		0.0500	0.0221	0.0132
	0.0391	0.0174	0.0086		0.0075	0.0031	0.0026
	0.0519	0.0235	0.0126		0.0516	0.0231	0.0122
	0.0526	0.0240	0.0129		0.0831	0.0373	0.0204

**Table 5:** Simulation results of magnetic dipole fitting errors under different inner diameters of loop.

	*E* *r_l_* = 4 mm	*E* *r_l_* = 6 mm	*E* *r_l_* = 8 mm		*E* *r_l_* = 4 mm	*E* *r_l_* = 6 mm	*E* *r_l_* = 8 mm
Coil1	0.0006	0.0013	0.0023	Coil2	0.0006	0.0013	0.0022
	0.0006	0.0013	0.0023		0.0005	0.0012	0.0021
	0.0007	0.0015	0.0027		0.0006	0.0014	0.0024
	0.0005	0.0012	0.0021		0.0005	0.0011	0.0020
	0.0006	0.0013	0.0022		0.0005	0.0012	0.0021

**Table 6:** Experimental localization accuracy for 18 coils and 11 OPM sensors.

Number of Sensor	Error of Position	Error of Angle
1	3.95 mm	1.44°
2	2.96 mm	1.58°
3	2.79 mm	3.26°
4	2.12 mm	3.35°
5	2.79 mm	4.29°
6	9.41 mm	9.02°
7	6.47 mm	10.09°
8	5.43 mm	1.88°
9	7.77 mm	9.99°
10	5.07 mm	3.38°
11	4.06 mm	9.72°
Mean ± SD	4.80 ± 2.20 mm	5.27° ± 3.46°

**Table 7:** Experimental results of sensor localization errors under optical orientation constraints (18 coils and 11 sensors).

Number of Sensor	Error of Position (±5%)	Error of Position (±1%)
1	3.95 mm	3.93 mm
2	2.96 mm	3.10 mm
3	2.79 mm	0.92 mm
4	2.12 mm	1.21 mm
5	2.06 mm	2.93 mm
6	7.68 mm	5.01 mm
7	6.88 mm	4.48 mm
8	4.59 mm	1.83 mm
9	7.27 mm	4.59 mm
10	4.30 mm	3.02 mm
11	4.63 mm	4.80 mm
Mean ± SD	4.48 ± 1.93 mm	3.26 ± 1.38 mm

## Data Availability

No new data were created or analyzed in this study.

## Appendix A

**Table A1:** Summary of Symbols and Parameters.

Symbol	Description	Unit
$V_{ICP}$	Vectorized point set after ICP alignment (OSI points transformed into MRI space for fine registration)	mm
$V_{OSI}$	Vectorized OSI point set (3D coordinates of extracted surface/marker points from the structured-light scanner)	mm
$R_{-}$	Rotation matrix	—
$t_{-}$	Translation vector in global coordinates	mm
$P_{-}$	3D point extracted from the OSI/marker cloud (coordinate of a single point)	mm
$\mu_{0}$	permeability of vacuum	4π × 10^−7^ H/m
$r_{s}$	position vector of the sensor	mm
$r_{c}$	position vector of coil	mm
$n_{s}$	unit vector of the sensor’s sensitive axis	—
$m_{c}$	Magnetic moment vector of the coil	A·m^2^
$r_{c,s}$	position vector of the sensor	mm
$B_{c,s}$	measured magnetic field	T
$r_{c}^{h}$	Parametric curve of the cylindrical helical coil	—
$r_{h}$	Inner radius of the cylindrical helical coil	mm
$h_{h}$	Wire diameter of the cylindrical helical coil	mm
$n_{h}$	number of turns of the cylindrical helical coil	—
$B_{d}$	Magnetic field of the same coil under the magnetic-dipole approximation	T
$B_{h}$	Magnetic field of the helical coil computed from the full Biot–Savart integral	T
E	quantify the fit between the dipole-model field and the ideal magnetic field	_

## Appendix C. Description of Workflow Modules

**Principal Component Analysis (PCA)**

PCA is applied to the raw OSM point cloud to remove global offsets and derive a decorrelated coordinate frame. The reconstructed surface may contain anisotropic distortions due to structured-light scanning; PCA re-centers the cloud, extracts the three principal orthogonal directions, and normalizes its orientation. This improves numerical stability and yields a consistent initialization for subsequent geometric processing.

**Feature Extraction**

For each OSM point, a set of geometric descriptors is computed, including surface normals, curvature estimates, and neighborhood statistics obtained via k-nearest-neighbor (k-NN) analysis. These features capture the local topology of the scanned head surface and are used to distinguish planar regions from curved areas. The resulting descriptors serve as inputs for clustering and local modeling.

**k-Means Clustering**

Based on the extracted geometric descriptors, the OSM point cloud is partitioned using k-means clustering. The goal is to group points with similar local geometric properties, producing coherent surface patches. This segmentation prevents mixing planar and non-planar regions and improves robustness for plane fitting and subsequent coarse alignment.

**Plane Fitting**

Each point cluster is fitted with a local plane using a least-squares optimization. The resulting planar approximations provide simplified local geometric models of the head surface. These planes are used to constrain the coarse alignment between the coil model and the OSM reconstruction and to eliminate unstable or poorly conditioned surface regions.

**SSIM-Based Sensor Localization**

In our pipeline, SSIM (Structural Similarity Index Measure) is integrated directly into the objective function for sensor localization. The implementation details of the projection rendering and SSIM-based similarity computation follow the method described in [34].

**Quaternion Rotation Representation**

To avoid singularities associated with Euler angles (e.g., gimbal lock), rotations are parameterized using unit quaternions. Quaternion-based updates ensure smooth, stable optimization during rigid-body alignment and improve convergence behavior. The final orientation obtained from coarse alignment and ICP refinement is represented as a normalized quaternion.
